# Blood flow-restricted resistance exercise alters the surface profile, miRNA cargo and functional impact of circulating extracellular vesicles

**DOI:** 10.1038/s41598-020-62456-3

**Published:** 2020-04-03

**Authors:** Jesper Just, Yan Yan, Jean Farup, Peter Sieljacks, Mette Sloth, Morten Venø, Tingting Gu, Frank Vincenzo de Paoli, Jens Randel Nyengaard, Rikke Bæk, Malene Møller Jørgensen, Jørgen Kjems, Kristian Vissing, Kim Ryun Drasbek

**Affiliations:** 1 Center of Functionally Integrative Neuroscience, Dept of Clinical Medicine, Aarhus University, Aarhus, Denmark; 2 Interdisciplinary Nanoscience Center, Aarhus University, Aarhus, Denmark; 3 Dept of Molecular Biology and Genetics, Aarhus University, Aarhus, Denmark; 4 Section for Sport Science, Department of Public Health, Aarhus University, Aarhus, Denmark; 5 Research laboratory for Biochemical Pathology, Dept of Clinical Medicine, Aarhus University, Aarhus, Denmark; 6 Dept of Biomedicine, Aarhus University, Aarhus, Denmark; 7 Dept of Biomedicine – physiology, Aarhus University, Aarhus, Denmark; 8 Dept of Clinical Medicine, Core Center for Molecular Morphology, Section for Stereology and Microscopy, Centre for Stochastic Geometry and Advanced Bioimaging, Aarhus University, Aarhus, Denmark; 9 Dept of Clinical Immunology, Aalborg University Hospital, Aalborg, Denmark; 10 Dept of Clinical Medicine, Aalborg University, Aalborg, Denmark

**Keywords:** Physiology, Molecular medicine

## Abstract

Ischemic exercise conducted as low-load blood flow restricted resistance exercise (BFRE) can lead to muscle remodelling and promote muscle growth, possibly through activation of muscle precursor cells. Cell activation can be triggered by blood borne extracellular vesicles (EVs) as these nano-sized particles are involved in long distance signalling. In this study, EVs isolated from plasma of healthy human subjects performing a single bout of BFRE were investigated for their change in EV surface profiles and miRNA cargos as well as their impact on skeletal muscle precursor cell proliferation. We found that after BFRE, five EV surface markers and 12 miRNAs were significantly altered. Furthermore, target prediction and functional enrichment analysis of the miRNAs revealed several target genes that are associated to biological pathways involved in skeletal muscle protein turnover. Interestingly, EVs from BFRE plasma increased the proliferation of muscle precursor cells. In addition, alterations in surface markers and miRNAs indicated that the combination of exercise and ischemic conditioning during BFRE can stimulate blood cells to release EVs. These results support that BFRE promotes EV release to engage in muscle remodelling and/or growth processes.

## Introduction

The beneficial adaptations to physical activity and exercise are known to involve paracrine and systemic mediators. In line with this, circulating extracellular vesicles (EVs) have recently been suggested to have an important metabolic impact on several physiological processes^1^. Extracellular vesicles have been shown to be released from a number of different cell types and detected in most body fluids including blood, urine, and saliva ^2–5^. They are protected by a lipid bilayer, range from 30–1000 nm in diameter, and can act as mediators of specific intercellular communication both locally and at distant sites through targeted cell receptor-ligand interactions and subsequent EV cargo release^6–8^. Extracellular vesicles secreted into the circulation carrying specific components may reflect the function and identity of the host cell. The magnitude of EV release, EV content and EV surface marker profile can be altered during different physiological conditions^9–11^. Interestingly, blood-derived EVs contain large amounts of microRNAs (miRNAs)^5^. These EV-packaged miRNAs have the ability to alter target cell gene expression by gene repression through complementary base-pairing with mRNAs and as such, EVs are particularly interesting from a functional point of view^12^. By analysing EV surface markers and content after exercise, one may be able to determine the tissues and cells that respond to exercise and make predictions on the possible functional role for the released EVs .

Muscle contractions inherent to exercise impose mechanical and metabolic stress on the muscle cells^13–15^; however, multiple other tissues are also affected by exercise including adipose tissue, bone marrow, and brain^16^. It is still unclear which mechanisms underlie the remote effect of exercise, but circulatory hormones, cytokines, or miRNAs loaded in EVs, represent likely candidates. This supports the notion that exercise can promote synthesis and release of molecules, such as EV-derived miRNAs, which may circulate to remote organs and potentially interact with a diverse range of target cells and infer physiological effects^17^.

Presently, only a few studies have conducted profiling of EVs and attempted to assess their functional impact in the context of exercise^11,18–20^. These studies report an increase in circulating EVs during and immediately after an acute bout of aerobic exercise, with the levels returning to baseline after a 1–3 hour recovery period. For instance, Whitham *et al*. conducted a characterisation of the EV proteome from subjects performing a moderate to high-intensity 1 hour cycling exercise bout^20^, observing an increase in classical EV proteins immediately after exercise that returned to base line after 4 hours of recovery. Secreted EVs were predominantly transferred to the liver and released their protein cargo there. In addition, muscle cells and other EV secreting tissues (e.g. adipose tissue) can release EVs into the circulation. The muscle-specific secretion of EVs has been found to increase acutely during aerobic exercise and account for 1–5% of the total EV pool^11^. However, exercise stressors are also suggested to have an impact on surrounding tissues such as the extracellular matrix (ECM), the microvascular network and/or components of the blood^21–23^. These tissues may also be important EV donors, especially in the circulatory system where most EVs originate from blood platelets, erythrocytes and monocytes^24^. Several studies have investigated the exercise-induced release of circulating miRNAs – both for free miRNA in plasma and for EV-enclosed miRNA. Most of these studies have focused on the characterisation of the classical muscle associated myo-miRs (miR-1, 206, 133a and 133b) in different exercise models (reviewed in^25^). While it is clear that exercise induces release of EVs and that these contain miRNAs, the physiological impact on exercise adaptation remains unknown. miRNAs have been shown to be involved in multiple processes, including the control of stem cell quiescence and proliferation^26^. For instance, acute exercise is known to stimulate stem cell proliferation, particularly in skeletal muscle, which could involve EV-delivered miRNAs (see below). Interestingly, one particular type of exercise, low intensity exercise combined with partial blood flow occlusion, Blood Flow Restricted Exercise (BFRE), has been reported to substantially induce muscle stem cell (MuSC, satellite cell) proliferation^27^. In addition to MuSCs, proliferation of a non-myogenic progenitor cell population (fibro-adipogenic progenitors, FAPs) has also been observed to increase following muscle damage^28^. In fact, studies have suggested FAP behaviour to be regulated by EVs released from MuSCs^29^. It is therefore tempting to speculate that EV-derived miRNAs could play a role in the BFRE-dependent induction of muscle stem cell proliferation.

BFRE resembles Remote Ischemic Conditioning (RIC, i.e., intermittent ischemia of an isolated extremity during resting conditions), which has been demonstrated to infer protective effects on remote tissues during lethal ischemic incidents^30–32^. We therefore hypothesised that miRNAs loaded in EVs and released during RIC or BFRE could induce stem cell activation and proliferation, thereby aiding tissue regeneration or adaptations to exercise. Moreover, since the ischemia-reperfusion of BFRE mimics that of RIC, the use of a BFRE model may also aid the delineation of how EV trafficking is related to exercise and reveal biomarkers of BFRE that could be exploited therapeutically.

Using BFRE as an experimental stimulatory mode, the purpose of the current study was therefore to investigate how BFRE affects the plasma EV profile, if BFRE EV origin can be deduced from these profiles, and explore if BFRE EVs carry a functional signal that can be transferred to distant tissues.

## Methods

### Subjects and study design

Study details on subject inclusion and exercise protocol have been described elsewhere^33^. The study was approved by the Ethical Committee for Region Midtjylland (ref. no. M-201430314) and conducted according to the standards of the Declaration of Helsinki. Written informed consent was obtained from all participants. Shortly, 9 recreationally active healthy men underwent a single BFRE bout. None had performed resistance training for the last 6 months and none had previous experience with BFRE. The subjects were instructed to refrain from strenuous activities 72 hours before the exercise session and avoid using pain-relieving medication. At least 7 days prior to the exercise session, the subjects unilateral knee extension 1-Repetition Maximum (1-RM) was tested and the subject’s anthropometrics were recorded (see Table 1).

**Table 1:** Anthropometrics of the included healthy participants (33).

	BFRE (*n* = 9)	*p* value
Age (years)	21 ± 0.6	0.73
Weight (kg)	74.7 ± 2.0	0.60
Height (cm)	183.2 ± 1.4	0.46
BMI (kg/m^2^)	23.3 ± 1.0	0.63
1RM leg extension (kg)	52.5 ± 6.6	0.11
MVC (Nm)	312 ± 15	0.25

BMI: Body mass index. MVC: maximal voluntary contraction torque. Mean +/− SEM.

### Training protocol

The BFRE bout consisted of five sets of knee extensions to volitional failure with 30% of 1-RM under partial blood flow restriction, which was achieved by placing a 135 mm wide curved pneumatic cuff (VBM Medizintechnik, Germany) around the proximal part of the thigh and inflating it to a pressure of 100 mmHg (Digital Tourniquet 9000, VBM Medizintechnik).

### Blood sampling and EV isolation

Blood samples were collected in EDTA vacuum tubes immediately before the BFRE bout and after 1 hour of post-exercise recovery. Plasma was isolated by centrifugation (1900*g* for 10 min, 5 °C), aliquoted, and stored at −80 °C. Haemolysis was estimated by absorbance of free haemoglobin at 414 nm on a Nanodrop 2000. The samples were considered haemolyzed if OD_414_ > 0.2.

Before EV isolation, plasma was thawed at 4 °C and centrifuged at 15,000 *g* for 15 min at 4 °C. For miRNA sequencing, EVs were purified by the miRCURY Exosome Isolation Kit – Serum and Plasma (Exiqon, Denmark) according to manufacturer’s instructions. Precipitation agent was added to 0.5 ml cleared plasma and incubated at 4 °C for 1 h. Precipitated EVs were then pelleted, resuspended in 0.3 ml Resuspension buffer and analysed by nano particle tracking analysis (NTA) (see below). For functional assays, EVs were purified from 1 ml cleared plasma by size exclusion chromatography (SEC) using qEV columns (Izon Bioscience, New Zealand) pre-equilibrated with dPBS (Lonza, Switzerland). Fractions of 0.5 ml were collected and EV content was analysed by tunable resistive pulse sensing (TRPS) (see below), while protein content was determined by absorbance at 280 nm on a Nanodrop 2000. EV fractions without protein contamination were pooled and concentrated by ultrafiltration (Amicon Ultra-2, 10 kDa, Merck Millipore, Germany) to a final volume of 100 µl. EVs in the concentrated samples were then analysed by NTA to allow direct comparison between the two EV purification methods.

### EV characterisation

Purified plasma EVs were diluted in PBS and subjected to nano particle tracking analysis (NTA) using the NanoSight LM10 (Malvern Instruments, UK) with a 405 nm laser. Measurements were performed 5 times with 60 s video captures of each sample and analysed using NanoSight NTA software (version 3.1, Malvern Instruments, UK) to determine sample concentration and EV size with corresponding standard error. A qNano Gold (Izon Bioscience) equipped with a NP100 Nanopore (Izon Bioscience) for TRPS was used to determine size and concentration of EVs in the fractions eluted by SEC. EV samples were analysed under identical settings - the same diluent, stretch (45 mm), pressure (10 Pa) and voltage (0.7 mV) as used for CPC100 calibration particles (Izon Bioscience). A minimum of 500 particles were measured for each sample. The EV concentration and size distribution were determined using the Izon control suite.

### Western blotting

Isolated EVs were tested for the presence of the EV markers TSG101 and Flot1 using western blotting. The concentrated EV fractions without protein contamination were lysed in SDS sample buffer (4x Laemmli sample buffer, BioRad, CA, USA) with 1.4-Dithiothreitol (DTT) for Flot1 and without DTT for TSG101 (non-reducing), separated by SDS-PAGE (12% Criterium TGX Precast Gels, BioRad) and transferred to mid-size transfer stacks (Trans-Blot Turbo, BioRad). Strips of the blots were blocked in 2% BSA-PBS and incubated with primary antibodies against flotillin 1 (1:500, 610821, BD Biosciences, CA, USA) and tsg101 (1:500, Ab83, Abcam, UK) followed by the secondary antibody, goat-anti-mouse-HRP (Z0334, Dako, CA, USA). Chemiluminescence (SuperSignal West Pico Chemiluminescent Substrate, Thermo Scientific) was recorded using a PXi4Touch (Syngene, UK).

### Transmission electron microscopy (TEM)

EVs were purified by precipitation and SEC as described above. Formwar film coated, 2 × 1 mm slot, were mounted in 50 µL drops containing purified EVs and left to rest for 20 min. The EVs were then fixed on the grid in 2% glutaraldehyde for 5 min and washed in PBS and distilled water. The grids were contrast stained with 0.5% uranyl acetate for 10 min and rinsed in distilled water. The EVs were visualised in a FEI Morgagni 268 transmission electron microscope equipped with a SIS III digital camera.

### EV Array

Microarray slides were produced for the EV Array as described by Jørgensen *et al*.^34^. Shortly, antibodies were printed on epoxy-coated slides (SCHOTT Nexterion, Germany) using a SpotBot**®** Extreme Protein Edition Microarray Printer with a 946MP4 pin (ArrayIt Corporation, CA, USA). Positive and negative controls were biotinylated human IgG (100 mg/mL) and PBS with 5% glycerol, respectively. A total of 40 anti-human antibodies, were used for production of the EV Array (see Suppl. Table S1). Antibodies were diluted in PBS with 5% glycerol and printed in triplicates at 200 mg/mL.

The EV Array was performed as described by Jørgensen *et al*.^35,36^, with modifications. In short, the microarray slides were initially blocked (50 mM ethanolamine, 100 mM Tris, 0.1% SDS, pH 9.0) prior to incubation with 10 µL plasma sample diluted (1:10) in wash-buffer (PBS/0.05% Tween®20). The incubation was performed in Multi-Well Hybridization Cassettes (ArrayIt Corporation) at RT for two hours followed by overnight incubation at 4 °C. A cocktail of biotinylated detection antibodies (anti-human-CD9, -CD63 and -CD81, Ancell, MN, USA) diluted 1:1500 was used to detect retained EVs using Cy5-labelled streptavidin (Life Technologies, MA, USA) diluted 1:1500. Scanning and spot detection was performed as previously described^36^.

### Small RNA isolation and next generation sequencing

Total RNA from purified EVs was isolated using miRCURY RNA isolation kit - plasma and serum (Exiqon) and eluted in 100 µl RNase free water. To concentrate RNA, 10 µl 3 M, pH 5.5 sodium acetate, 250 µl pre-chilled 99% ethanol and 1 µl Glycoblue (Ambion, MA, USA) were added and incubated at −20 °C overnight. RNA was pelleted by centrifugation at 16,000 *g* for 10 min at 4 °C and re-suspended in 6 µl RNase free water. The small RNA library of EVs was constructed using Illumina TruSeq Small RNA Sample Prep Kit (Illumina, CA, USA) by adding 5 µl of total RNA as input. Due to lower RNA input compared to the standard protocol, the amount of adaptors was reduced to 1/10, the other reagents were reduced by half, and PCR cycles were increased from 12 to 15. The library was purified in the size range 140bp-160bp by Pippin Prep (Sage Science, MA, USA). The size and purity of the cDNA libraries were validated on a 2100 Bioanalyzer High Sensitivity DNA chip (Agilent, CA, USA) and the concentration was quantified using KAPA Library Quantification Kit (KAPA biosystems, Switzerland). The libraries were pooled as required and sequenced on the Illumina Nextseq 500 instrument by Exiqon.

### NGS data analysis

Trim galore was used to trim low-quality ends from raw reads in addition to adaptor removal (Babraham Bioinformatics). A Phred33 cut-off score of 20 was applied before adaptor trimming, and all sequences below 16 bp were subsequently removed. The clean reads were then analysed by the nc-PRO-seq pipeline using the standard settings^37^. Shortly, the reads were aligned to the HG19 genome with Bowtie and overlaps between read alignments and genomic annotations (Rfam, RepeatMasker and miRbase) were investigated using BEDTools. Due to miRNA-end heterogeneity, miRNA annotations were extended 2 bases in both upstream and downstream regions. The raw miRNA counts were then normalised and tested for differential expression (DE) using the R Bioconductor package DESeq2, implementing a model design testing for differences between pre and post BFRE while correcting for inter-person variability^38^. DE miRNAs were considered significant with an FDR adjusted p-value <0.05, a log2 fold change > ±0.5 and a base mean count >100. The rlog function of DESeq2 was called to transform the miRNA counts to the log2 scale, which was used as input for principal component analysis by the plotPCA function of DESeq2 and ggplot2^39^. Polygons consisting of the smallest convex sets that contained the pre- or post-BFRE samples were calculated and added to the PCA plot. The differentially expressed miRNAs were presented in a volcano plot created in the R Bioconductor package EnhancedVolcano (Blighe K (2019). *EnhancedVolcano: Publication-ready volcano plots with enhanced colouring and labeling*. R package version 1.2.0).

### EV Array data analysis

The corrected log2 transformed intensity values were used as input for differential expression analysis. The analysis was performed using the R Bioconductor package Limma where a model design was implemented to test for differential expression between pre and post BFRE, while correcting for inter-person variability^40^. A surface marker was considered differentially expressed when the calculated p-value was <0.05.

### Target prediction and enrichment analysis

For target prediction, DE miRNAs were used as input in the miRnet database that integrate high-quality miRNA-target interaction from several databases^41^. A miRNA-target interaction network was generated, and the gene target names of all miRNAs were then used as input in gene set over-representation analysis by the R Bioconductor package XGR^42^. Functional pathway analysis on all pathway gene sets (MsigdbC2CPall) including KEGG, BioCarta, REACTOME and PID was assessed with a minimum overlap of 5 target genes. The significance of the overlaps was determined using a cut-off of FDR adjusted p-value <0.05 and visualised by XGR according to fold enrichment.

### Fluorescence activated cell sorting (FACS)

To examine the effect of EVs on human stem cells we FACS isolated primary cells from adult human skeletal muscle. Muscle tissue was obtained from the m. rectus abdominis during aortic aneurism surgery with patient consent and in accordance with the acceptance from the Local Ethical Committee of Central Denmark Region (ref no. 54952). Muscle was transported to the laboratory within 15–20 min in ice-cold wash buffer [Hams F10 incl. glutamine and bicarbonate (Sigma, Sigma-Aldrich, Denmark), 10% Horse serum (Gibco, ThermoFisher Scientific, MA, USA), 1% Pen/strep (Gibco)]. Upon arrival, the muscle biopsy was initially dissected free of visible tendon/connective tissue and fat. The biopsy was then divided into pieces of 0.5–0.8 g and briefly mechanically minced with sterile scissors. The muscle slurry was then transferred to C-tubes (Miltenyi Biotec, Lund, Sweden) containing 8 ml wash buffer, 700 U/ml Collagenase II (Worthington, Lakewood, NJ, USA) and 3.27 U/ml Dispase II (Roche Diagnostics, Basel, Switzerland). Mechanical and enzymatic muscle digestion was then performed on the gentleMACS with heaters (Miltenyi Biotec) for 60 min using a skeletal muscle digestion program (37C_mr_SMDK1). When digestion was complete 8 ml wash buffer was added to the single cell solution and filtered through a 70 µm cell strainer and washed twice to collect any remaining cells. The suspension was centrifuged at 500 *g* for 5 min. The cell pellet was resuspended in freezing buffer (StemMACS, Miltenyi Biotec) and stored 1–2 weeks at −80 °C.

Approximately 1.5 hour before FACS, cells were thawed and 10 ml of wash buffer was added. The solution was centrifuged at 500 *g* for 5 min and the supernatant removed to clear the freezing buffer. The cells were then resuspended in wash buffer and incubated in MACS human FcR blocking solution (20 µl/sample, Miltenyi Biotec) and primary antibodies against CD45-FITC (12 µl/sample, Clone 5B1, MACS Miltenyi Biotec), CD31-FITC (4 µl/sample, Clone REA730, Miltenyi Biotec), CD90-PE (1:200, Clone 5E10, eBioscience, San Diego, USA), CD56-BV421 (1:100, Cat No.562752, BD Bioscience, San Jose, CA, USA), CD34-APC (20 µl/sample, clone 581, BD Bioscience) in darkness at 4 °C for 40 min. Propidium iodide (PI, 10 µl/sample, BD Bioscience) was added to the solution to exclude dead cells during sorting. The suspension was finally washed and filtered through a 30 µm filter to remove any remaining debris/aggregates. The solution was centrifuged at 500 g and resuspended in sorting buffer (PBS, 0.2% BSA, 1 mM EDTA). Non-stained cells and single-color controls were prepared in combination with the primary (full colour) samples. To ensure bright single-color controls for compensation, compensation beads (eBioscience, ThermoFisher Scientific) was utilized. Cell sorting was performed using a FACS-AriaIII cell sorter (BD Bioscience) with 405 nm, 488 nm, 561 nm and 633 nm lasers. A 100 µm nozzle was utilized to lower pressure/stress on the cells as well as prevent clogging. Gating strategies were optimized through multiple earlier experiments, which included various unstained, single colour, fluorescence minus one controls (FMO controls for CD34, CD90 and CD56, Suppl. Fig. S1) and full colour samples. Cells were sorted into 4 °C cooled collection tubes containing wash buffer. MuSCs were sorted as CD56^+^CD34^−^CD45^−^CD31^−^PI^−^ and FAPs were sorted as CD34^+^CD90^+^CD56^−^CD45^−^CD31^−^PI^−^. The purity of the population was checked following the sort by re-running the samples which yielded >96% pure populations and later by immunocytochemistry (ICC) when cells were plated.

### Cell culture and EdU detection

Immediately following sorting, MuSCs and FAPs were plated at approximately 1 × 10^4^ cell/cm^2^ in 0.5% gelatin coated flasks or wells and kept at 37 °C and 5% CO_2_. Both MuSCs and FAPs were plated in growth media (Bio-AMF 2, Biological Industries, Israel). When reaching 50–70% confluence the cells were re-plated at 3 × 10^4^ per well in growth media in 8-well chamber slides (Falcon, ThermoFisher Scientific) initially coated with Poly-D-Lysine and immediately before plating coated with 0.5% gelatin. Cells were either fixed for ICC after three days to confirm identity by staining or subjected to proliferation experiments. For proliferation experiments the cells were washed in PBS once and serum starved for 18 hours in Opti-MEM (Gibco) three days post plating. Following starvation, the media was replaced with Opti-MEM containing 10 µM EdU and 20% PBS with resuspended EVs (pre or post BFRE) or PBS without EVs. Cells were incubated for 24 hours and then fixed in 4% paraformaldehyde.

For the differentiation assay MuSCs were isolated using FACS and plated in a 96-well half area tissue culture plate (Corning, Sigma) coated with extra-cellular matrix (E1270, Sigma). Cells were initially plated at a density of 1 × 10^4^ and allowed to grow to 95% confluency. The media was then switched to differentiation media containing 50 µl EVs (pre- or post-BFRE in PBS) or control (PBS) and 100 µl DMEM (high glucose, Sigma) + 2% FBS (exosome depleted, Gibco) + 1% pen/strep (Gibco). After two days, the differentiation was stopped by fixing the cells in 4% PFA.

EdU was detected using the Click-it imaging kit (Invitrogen, MA, USA) and Hoechst 33342 was added to stain the nuclei. Total nuclei and EdU positive nuclei were imaged on a Leica DM2000 fluorescent microscope and a Leica Hi-resolution Colour DFC camera (Leica, Germany) at 10x and analysed in the Leica Application Suite (LAS, Leica) and ImageJ (imagej.nih.gov) using the cell counter plugin.

### Immunocytochemistry

MuSCs and FAPs were stained using ICC to confirm the identity of the cells while MuSCs in the differentiation assay was stained for myosin heavy chain (MyHC). After blocking for 20 min in 10% goat serum or FBS (for PDGFRa detection) with 0.5% Triton-X-100, the cells were stained with antibodies against Desmin 1:200 (D94F5, Cell Signaling, MA, USA) and MyoD1 1:100 (5.8 A, Dako) or MyHC (1:5 (MF20, Developmental Studies Hybridoma, IA, USA) for MuSCs or PDGFRa 1:100 (AF-307-NA, R&D Systems, MN, USA) for FAPs in PBS (1% BSA). Appropriate secondary antibodies in 1:500 (Alexa-fluor 647 goat-anti-mouse (A21236), Alexa-fluor 568 goat-anti-mouse (A11004), Alexa-fluor 488 goat-anti-rabbit (A11008), Alexa-fluor 568 donkey-anti-goat (A11058), Invitrogen) were used together with DAPI as nuclei staining before mounting. Images were obtained as described above. Images for MuSC differentiation was obtained using an EVOS 7000 (Invitrogen) fully automated imaging system. Each well was entirely imaged to ensure comparability between wells. Images were analysed in MyoCount open access software in Matlab^43^ to ensure objective evaluation. The Fusion index was calculated as the number of nuclei in MyHC^+^ myotubes containing more than three nuclei divided by total number of nuclei. Myotube size was quantified as the percentage of MyHC^+^ area relative to the total area of each well.

### EV fluorescent labelling and EV uptake

The EVs were isolated by SEC as described above and labelled using the PKH67 Green Fluorescent Cell Linker Kit (Merck). In short, 3*10^10^ EVs were diluted in Diluent C (Merck) to a volume of 1 ml and 10 µl PKH67 dye was then gently mixed with the EVs for 5 min at room temperature. Next, dPBS (Lonza) was added to a final volume of 10 ml and the EVs were separated from free dye by SEC using a qEV10 column (Izon bioscience). For the control staining, the exact same labelling steps were carried out while omitting EVs. For the uptake analysis, MuSCs and FAPs were plated at 10.000 cells/well in µ-Slide VI 0.4 slides (Ibidi) and maintained as described above. The labelled EVs were mixed 1:1 with Opti-MEM and incubated with MuSCs and FAPs for 24 hours. The cells were then washed in dPBS (Lonza) and fixed in 4% paraformaldehyde. DAPI was added to stain the nuclei. The labelled EVs and nuclei were imaged on a Leica fluorescent microscope (DM6000 B, Leica) and an Olympus DP72 digital colour camera (12.8 megapixel, Olympus, Denmark).

### Statistics

The statistical tests used for the EV Array data analysis, NGS data analysis and enrichment analysis have been described in the relevant sections above. For bar plots, data are shown as the mean ± SEM. For the proliferation assay (EdU incorporation) and differentiation assay, the control group was compared to the naïve pre-BFRE EV group by a parametric, unpaired students t-test, while the comparison between intervention groups were compared by a parametric, paired students t-test. The statistical analysis was performed by statistical software (GraphPad Prism 7.0, Graphpad Software Inc., CA, USA).

## Results

### The surface marker profile of circulating EVs change after BFRE

To shed light on the possible origin and functional impact of BFRE EVs, human plasma from healthy volunteers was analysed using the EV Array, which utilises a multiplex immunoaffinity capture format to analyse the expression of surface markers on circulating EVs. A priori, 40 surface markers were chosen and included in the assay based on the expected cell-origin and markers involved in physiological adaptations associated with exercise (for the full list, see material and methods, EV Array). Only plasma samples unaffected by haemolysis was included (free haemoglobin OD_414_ < 0.2, Suppl. Fig. S2). Plasma from subjects with pre/post BFRE sample pairs was used as starting material for the EV Array (n = 6). Differential expression analysis revealed that four surface markers were up-regulated (Integrin alpha 2b (ITGA2B/CD41), Neural cell adhesion molecule (NCAM), Interleukin-2 receptor alpha chain (IL2RA/CD25) and programmed cell death 6 interacting protein (PDCD6IP/ALIX), while one was down-regulated (Flotillin 1) after BFRE (Table 2). Furthermore, the levels of the canonical EV markers CD9, CD63 and CD81 were unchanged after BFRE, indicating that no overall change in EV quantity was observed one hour post BFRE (Table 2). Hence, a change in the surface protein expression and/or membrane incorporation pattern of the EV donor cells likely caused the observed surface marker change.

**Table 2:** EV Array analysis of plasma EV surface marker expression before and after BFRE.

EV surface marker	% change to pre	P-Value
Integrin alpha 2b/CD41	36	0.00019
Flotillin 1	−24	0.00019
NCAM	25	0.0026
Alix/PDCD6IP	18	0.024
IL2RA/CD25	21	0.049
CD81	6	0.41
CD63	5	0.50
CD9	1	0.88

Antibodies against 40 surface proteins were printed on epoxy-coated glass slides and used to capture EVs present in plasma. A cocktail consisting of antibodies against the canonical EV surface proteins CD9, CD63 and CD81 coupled with fluorescent probes were used for detection. Five surface markers changed expression after BFRE. Four were up-regulated: CD41, NCAM, Alix, CD25; while one was down-regulated: Flotillin-1. No change in the canonical EV markers CD9, CD63, CD81 was observed.

### Neither concentration nor size distribution of isolated circulating EVs changes one hour after BFRE

**Figure 1:**
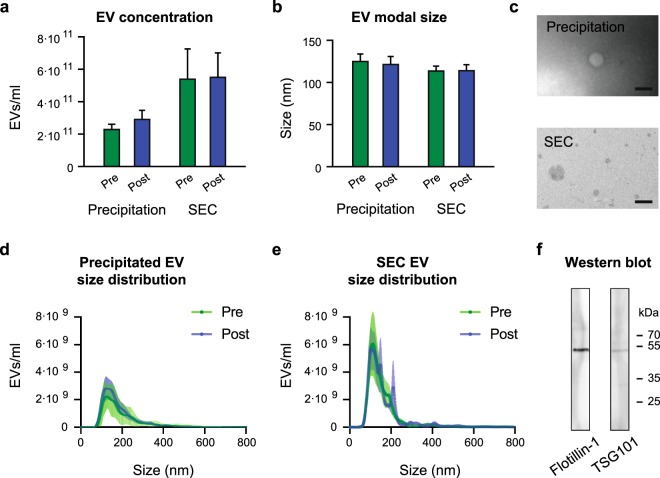
Characterisation of EVs purified from plasma collected before and after BFRE. Using the NTA technology, the EV concentration (**a**) and size (**b**) were determined for the two purification methods, precipitation using the miRCURY Exosome Isolation Kit (0.5 ml plasma) or size exclusion chromatography (SEC) using qEVs (1 ml plasma) (mean +/− SEM, n = 6). Transmission electron microscopy (TEM) images of EVs (**c**), scalebar 200 nm. NTA generated size distribution of purified EVs using precipitation (**d**) and SEC (**e**). Full length Western blotting strips validating the presence of the EV markers Flotilin-1 and TSG101 (**f**).

EVs from subjects with pre/post BFRE sample pairs were isolated by polymer-based precipitation and size exclusion chromatography (SEC) (n = 6 for each group). For SEC, the EV elution profile revealed that EVs devoid of protein contamination were present in fraction 6–9 (Suppl. Fig. S3). These EV fractions were pooled and used for downstream analysis. The isolated EVs were then characterised by nanoparticle tracking analysis (NTA), TEM, and western blotting (Fig. 1) and we found no significant change in concentration or size of the isolated EVs after BFRE using either isolation method (Fig. 1a,b), which was in agreement with the EV Array above. Thus, the increase in EV concentration previously reported immediately after exercise, and also seen 5 min after BFRE (our own unpublished data), returns to baseline within 1 hour in this exercise modality. EVs isolated by SEC had a modal size of approx. 115 nm (Fig. 1b,e), while EVs isolated by polymer-based precipitation had a modal size of 125 nm. Additionally, a small subset of larger vesicles,> 200 nm, were also isolated by this method (Fig. 1b,d). EV identity was further confirmed by TEM, and the presence of the EV markers Flotilin-1 and TSG101 was verified by western blotting (Fig. 1c,f). Thus, both EV isolation methods yielded EVs with similar characteristics.

### The miRNA content of EVs was altered after BFRE

**Figure 2:**
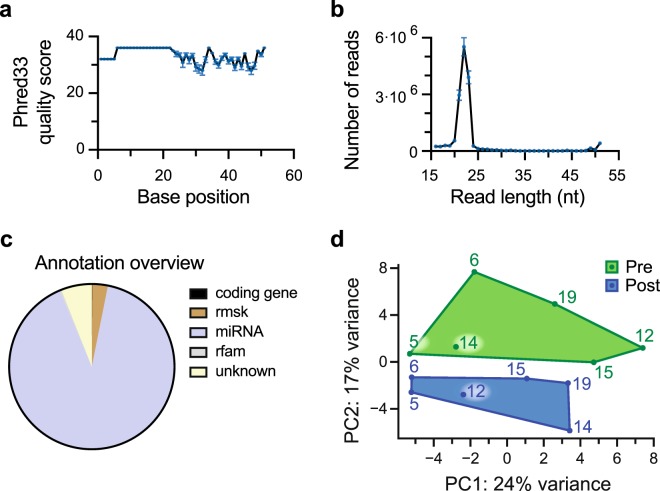
Next generation sequencing of small non-coding RNAs purified from EVs. The quality score assigned to each base position after quality and adaptor trimming. (**a**) The read length distribution (**b**) shows a peak from 20–25 nucleotides. Annotation overview of reads (**c**), with approximately 80% of the reads mapping to miRNAs. PCA plot based on the counts of all detected miRNAs. (**d**) A green (pre-samples) and blue (post-samples) polygon denotes the smallest space to contain the pre and post samples, respectively. Rmsk: RepeatMasker, rfam: Database of functional non-coding RNA families.

**Figure 3:**
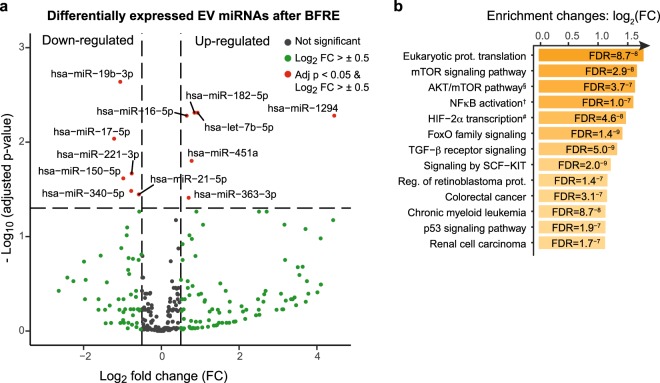
Differentially expressed plasma EV miRNAs after BFRE presented as a volcano plot. (**a**) The miRNA counts from NGS were normalised and tested for differential expression (DE) using the R Bioconductor package DESeq2, implementing a model design testing for differences between pre and post BFRE while correcting for inter-person variability. DE miRNAs (red) were considered significant with an FDR adjusted p-value <0.05, a log2 fold-change > ±0.5 and a base mean count >100. Under these assumptions, 12 miRNAs were differentially expressed (6 were up-regulated and 6 were down-regulated). Pathway enrichment analysis (**b**), showing top 15 enriched pathways from KEGG, REACTOME, BioCarta and PID collected in the MsigdbC2Pall pathway gene sets with FDR < 0.05. ^§^Skeletal muscle hypertrophy is regulated via AKT/mTOR pathway. ^†^NFkB activation by Non-typeable Hemophilus influenzae.

After EV surface marker characterisation, we isolated the small RNA content of the precipitated vesicles from each subject (n = 6) before and after BFRE for next generation sequencing. The quality and read length distribution of the reads were assessed with a peak at 20–25 nt (Fig. 2a,b). The reads were then aligned to the human genome (hg19) and the annotation overview showed that 80% of the mapped reads were recognised as miRNAs (Fig. 2c). The remaining 20% included siRNA, rRNA, snoRNA, tRNA and piRNA. The raw miRNA counts were normalised as described in the Bioconductor package DESeq2, and a PCA plot based on the counts of all detected miRNAs for each subject showed a well-defined separation between pre and post samples (Fig. 2d). Inter-person variability was also observed. Differential expression (DE) analysis revealed that the expression of 12 EV miRNAs were significantly altered after BFRE with 6 up-regulated miRNAs while 6 were down-regulated (Fig. 3a).

### BFRE EVs activate and induce S-phase entry in human primary MuSCs and FAP cells

To pinpoint the collective functional impact of the DE miRNAs, experimentally validated gene targets were retrieved from miRBase (Suppl. Table S2). Several genes involved in cell cycle control and cell proliferation were found to be targeted by the highest number of miRNAs e.g. Cyclin D2, Cyclin D1, p21, Insulin like growth factor 1 receptor, c-myc, Foxo3, MAPK, and p53 (Suppl. Table S2). In addition, using all predicted gene targets of the DE miRNAs for functional enrichment analysis, we primarily found potential cell cycle and well-known exercise/muscle related pathways (Fig. 3b), including mTOR signalling, AKT/mTOR signalling in muscle hypertrophy, HIF-2α, FoxO family signalling, TGFβ receptor signalling, SCF-KIT, p53 signalling, and MAP kinase signalling.

Since several of the predicted targets for DE miRNAs were related to cell cycle and cell activation, we decided to examine if the post-BFRE EVs had an impact on human stem cell proliferation. We focused our attention on stem cells localised in skeletal muscle since this is the major organ affected by BFRE. Furthermore, the position of these cells within the muscle tissue allow for both local and systemic communication. For this assay, the EVs were isolated using size exclusion chromatography, as the polymer-based method used for EV miRNA-seq unfortunately caused the cell culture medium to solidify over the course of the experiment. This was likely due to leftover polymers, or other compounds, in the precipitation buffer. As described above, both EV isolation methods yielded EVs with similar characteristics.

**Figure 4:**
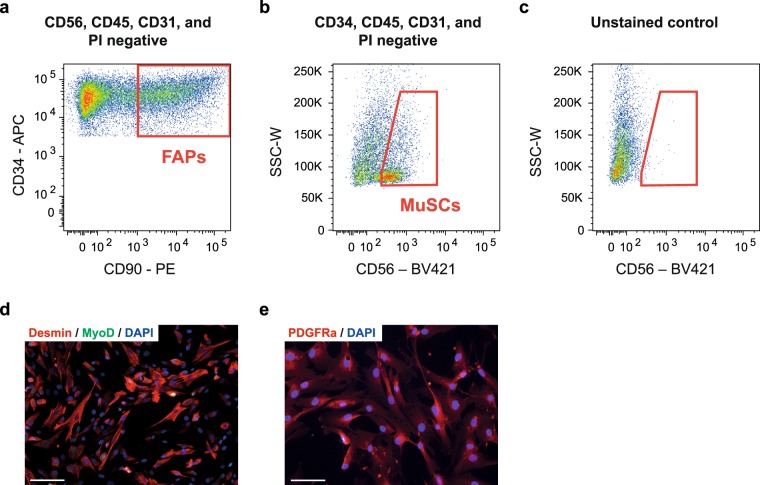
FACS plots showing the sorting strategy to purify human skeletal muscle stem cells (MuSCs) as CD56^+^CD34^−^CD45^−^CD31^−^PI^−^ (**a**) and fibro-adipogenic progenitors (FAPs) as CD34^+^CD90^+^CD56^−^CD45^−^CD31^−^PI^−^ from fresh muscle biopsies (**b,c**). Immunocytochemistry confirming that sorted MuSCs are myogenic (MyoD^+^-green, Desmin^+^-red (d)) and the FAPs express PDGFRa (red (**e**)). Nuclei were stained with DAPI (blue).

**Figure 5:**
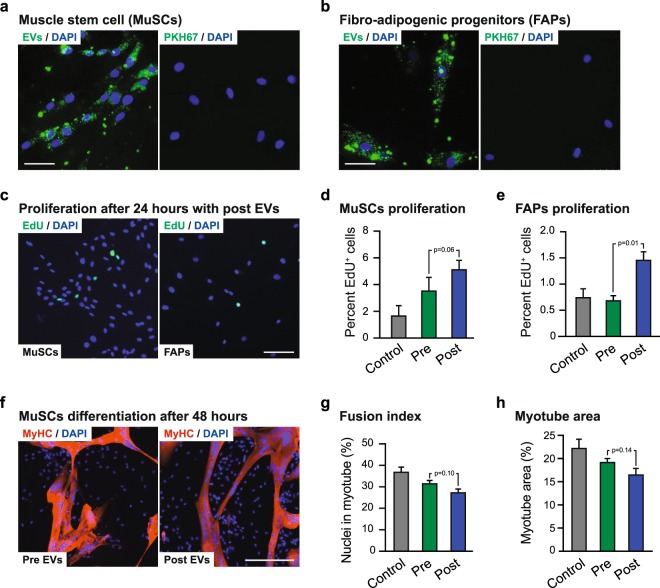
Fluorescently labelled post-BFRE EVs (green) were taken up by MuSCs (**a**) and FAPs (**b**) during 24 hours of incubation. Only a small amount of signal was observed in the negative control (PKH67, right panel). Scalebar = 50 µm. Proliferation after 24 hours was estimated based on EdU incorporation (**c**) in MuSCs (**d**) and FAPs (**e**) incubated with either pre- or post-BFRE EVs or non-EV control (PBS). MuSC differentiation into multi-nucleated myotubes after 48 hours when cultured with the pre- or post-BFRE EVs (**f**). Differentiation estimations were based on the fusion index (**g**) (percentage of nuclei in MyHC^+^ (red) myotubes containing more than three nuclei) and the myotube area (**h**). (1.0·10^10^ EVs were added per well) (mean +/− SEM, n = 6).

Human MuSCs and FAPs were isolated from skeletal muscle by FACS (Fig. 4a–e) and showed a clear accumulation of fluorescently labelled post-BFRE EVs after 24-hours incubation (Fig. 5a,b). These cells were then cultured for 24 hours with pre- or post-BFRE EVs isolated by SEC from each subject (n = 6) and EdU incorporation was analysed to assess cell proliferation (Fig. 5c–e). Proliferation in FAPs were significantly increased after post-BFRE EV administration compared to pre-BFRE EVs (p = 0.013). In addition, when comparing the effect of pre- and post-BFRE EVs on MUSCs, we observed an increase in proliferation that did not reach significance (p = 0.062). In contrast, administration of pre-BFRE EVs did not affect proliferation in either cell type as compared with the non-EV control (PBS) (MuSC: p = 0.23; FAPs: p = 0.75) suggesting that the post-BFRE EVs carry specific proliferation signals.

To further describe the functional impact of post-BFRE EVs on MuSCs, we investigated the effect on MuSC differentiation into multi-nucleated myotubes. The MuSCs were cultured with the pre- or post-BFRE EVs for 48 hours before assessing the fusion index (percentage of nuclei in MyHC^+^ myotubes containing more than three nuclei, Fig. 5f–h). While not significant, a tendency towards a lower fusion index was observed for the post-BFRE EVs compared to the pre-BFRE EVs (p = 0.10) (Fig. 5h). Furthermore, it was suggested that plasma EVs (pre- and post-BFRE EVs) in general may reduce the fusion index when compared to the non-EV control (PBS) (p = 0.09 and p = 0.01, respectively). Analysis of the myotube area further supported this, as a tendency towards decreased myotube area was observed following administration of post-BFRE EVs compared to pre-BFRE EVs (p = 0.14) (Fig. 5h).

## Discussion

EVs are increasingly recognized as paracrine systemic mediators of exercise adaptations and remote organ conditioning^20,44^. Here, we used a low intensity ischemic resistance exercise model (BFRE) to investigate changes in the circulating EV surface marker content and miRNA profile and to investigate functional impact of these changes. Using a hypothesis-driven phenotyping platform, the EV Array, combined with unbiased miRNA sequencing, we analysed the EV surface profile and EV miRNA cargo in plasma from healthy volunteers, before and 1 hour after BFRE. We identified a BFRE-induced change in EV surface marker expression and differential expression of several EV miRNAs. Furthermore, miRNA target prediction and* in silico* functional enrichment analysis showed association to genes involved in cell cycle control and cell growth. Accordingly, we demonstrate that post-BFRE EVs possess the ability to activate and induce s-phase entry in both MuSCs and FAPs isolated from human skeletal muscle.

Four vesicle surface markers increased following BFRE (ITGA2B, NCAM, IL2RA, ALIX), while one marker was decreased (Flot1). ITGA2B is mainly expressed in the bone marrow by hematopoietic cells (myelopoietic and erythropoietic cells)^45^ and is essential in the coagulation cascade, supporting earlier reports that exercise-induced EVs can promote coagulation^46,47^. NCAM is expressed in brain and peripheral neuronal cells, glial cells, NK cells and endothelial cells. Interestingly, MuSCs also express this marker abundantly, likely in order to regulate interactions between neurons and muscle^48^. IL2RA is associated with the bone marrow and immune system with expression observed in hematopoietic cells, lymph nodes, tonsil and spleen and more specifically found on activated T-cells, B-cells and myeloid precursors^49^. Both PDCD6IP (Alix) and Flot1 are ubiquitously expressed and both have been shown to be involved in EV formation, secretion and/or sorting^50^. Alix has also been shown to be involved in miRNA packaging into EVs^51^. Taken together, the response seems to be pronounced for cells in the blood, bone marrow, or cells closely associated hereto, which is in line with another study that showed that lymphocytes, monocytes, platelets, endothelial cells and antigen presenting cells released EVs after an incremental cycling test^44^. In support of this, the differentially expressed miRNAs are also expressed by cells in the circulation and for the up-regulated EV miRNAs it is especially pronounced that CD235a-positive cells, erythrocytes, seem to be major contributors of miR-182 and miR-451a (Suppl. Fig. S4)^52^. The up-regulated miRNAs were further characterised by an expression atlas of miRNAs, and was found enriched mainly in hematopoietic cells, leukocytes and neutrophils^53^.

From the data reported in this study it appears that the combination of exercise and ischemic conditioning during BFRE have an impact on blood cells, which causes them to release EVs. We did not observe increases in specific muscle associated myo-miRs. This is in concordance with a recent study that found no change in circulating myo-miRs when comparing pre-exercise to post-exercise in three different resistance exercise protocols^54^. Muscle cells can release EVs into the circulation and this secretion has been reported to increase acutely during exercise^11^, but these muscle-specific EVs only account for 1–5% of the total EV pool. Our results therefore indicate that the hypoxic environment and the shear stress associated with muscle contractions in BFRE affect blood cells and bone marrow. From a functional point of view, this would make sense since the vast majority of plasma EVs have been shown to originate from platelets, erythrocytes and monocytes^24^.

Circulating miRNAs associated with exercise and ischemic conditioning have previously been characterised^25,55^. In the majority of these studies, pre-selected miRNAs isolated from plasma were analysed, including miRNAs in EVs, miRNAs in lipid complexes or free complexes of miRNAs and the protein Argonaute-2^25^. The characterisation of miRNAs following exercise has been dominated by the classical myo-miRs; however, the abundance of myo-miRs present in the blood after exercise seems to be limited, in agreement with our observations. Thus, as opposed to their relevance as muscle homeostasis biomarkers, the systemic impact of circulating myo-miRs is likely minor. That said, we do see an overlap between the miRNAs found in this study and previous reports in the literature. For different modalities of exercise, alterations in expression of miR-182–5p, 451a, 16–5p, 363–3p, 21–5p have previously been reported^54,56–59^. The hypoxia-inducible miR-182–5p is of specific interest for BFRE because of its up-regulation, abundance and previously described packaging into EVs^60,61^. Furthermore, this miRNA has been shown to enhance HIF1α signalling, to protect cardiomyocytes from hypoxia-induced apoptosis, modulate glucose utilization in muscle and increase angiogenesis *in vitro*^60,62–64^. With 12 differentially expressed miRNAs in post-BFRE EVs, we focused on the combined effect of these changes. Therefore, all differentially expressed miRNAs were used as input for functional enrichment analysis. The top 10 enriched pathways illustrate that genes involved in cell cycle and cell proliferation are targeted by several of the miRNAs that show altered expression following BFRE (Fig. 3).

Considering that exercise in general and perhaps BFRE in particular can stimulate proliferation of muscle stem cells, we tested the impact of post-BFRE EVs on stem cell proliferation. As isolation of EVs by precipitation was not compatible with cell culturing, we implemented size exclusion chromatography for EV purification of these studies. While EV chacteristics were similar for the two isolation techniques, we can not rule out that other differences may exist. Initially, we focused our attention on MuSCs, since these are well known to respond to exercise. Interestingly, we found that activation and entry into S-phase, as evaluated by EdU incorporation of FACS isolated primary human MuSCs, was enhanced by incubation with post-BFRE EVs. Similar findings emerged for FACS-isolated FAPs, a non-myogenic mesenchymal progenitor cell from human skeletal muscle, suggesting that the EV-induced proliferation effect is not confined to MuSCs. On the other hand, myogenic cell-cycle specific targets such as MyoD1 was not a predicted target of the differentially expressed miRNAs, which further support a more general proliferative effect of the post-exercise EVs. In agreement with this, we did not observe a significant difference between pre- and post-exercise EVs on MuSC differentiation. However, we did note a tendency of post-BFRE EVs towards reducing MuSC differentiation compared to pre-BFRE EVs. The enhanced proliferation and possible reduced differentiation mediated by post-BFRE EVs is in agreement with the effect of BFRE training on MuSC proliferation *in vivo*^27^. Thus, EVs may mediate or at least support the initial proliferation of MuSCs and FAPs post-BFRE and prevent differentiation to ensure expansion of the stem cell pool before cells initiate differentiation. This is supported by the increased MuSCs content observed during BFRE intervention and the subsequent reduction in MuSC content post training reported by Nielsen *et al*.^27^. In relation to MuSC differentiation we report a general tendency towards a reduction in MuSC differentiation induced by plasma EVs. In contrast to MuSCs, FAPs are present in multiple tissues (e.g. cardiac and adipose tissue), so the effect of post-BFRE EVs on FAPs is likely not restricted to skeletal muscle. This is intriguing as BFRE, similarly to RIC, may have the ability to trigger remote organ effects. As we see a clear accumulation of fluorescently labelled BFRE EVs in both cell types, our findings suggest that one mechanism behind this could be EV-induced activation of stem and progenitor cells. It has recently been shown that remote injury can prime stem and progenitor cells in non-injured organs^65^. In this “alert” stage, as the authors characterised it, the stem and progenitor cells were more likely to be activated and regeneration was enhanced when stem cells had been previously stimulated (alerted)^65^. The alert phenotype was dependent on signalling through the mTOR pathway shown by conditional deletion of Raptor. Interestingly, the prediction analysis performed in our study also points to mTOR signalling, suggesting that post-BFRE EVs may have the capacity to activate this pathway. Thus, we speculate that miRNAs in EVs released following BFRE may have a similar effect and prime (alert) stem cells for activation as well as fully activate some cells. However, other EV components like surface proteins, protein cargo or lipid cargo might also contribute to the increased proliferation in these cells.

In conclusion, we provide evidence that ischemic exercise conducted as BFRE can promote changes in the EV surface profile and miRNA content. Our data indicate EVs are predominantly derived from blood cells and bone marrow after BFRE. Furthermore, miRNA target prediction and *in silico* functional enrichment analysis show significant association to key proteins and pathways involved in cell cycle control and cell growth. Accordingly, post-BFRE EVs had the ability to activate and induce s-phase entry in MuSCs isolated from skeletal muscle tissue. This study supports the notion that EVs are dynamic nano-particles capable of mediating effects of physical stress-induced adaptations – even remotely when released in the blood. Pinpointing the exact mechanism of these changes could provide novel insight and possibly provide targets for therapeutic exploration.

## Supplementary information

Supplementary information
