# Supplementary material for: Blood flow-restricted resistance exercise alters the surface profile, miRNA cargo and functional impact of circulating extracellular vesicles
Source: Sci Rep. 2020 Apr 3;10:5835. doi: 10.1038/s41598-020-62456-3 (PMC7125173; doi:10.1038/s41598-020-62456-3)
Supplement: Supplementary file 1 — Supplementary information [file 41598_2020_62456_MOESM1_ESM.pdf]

Supplementary Tables and Figures

Title

Blood flow-restricted resistance exercise alters the surface profile, miRNA cargo and functional impact of circulating extracellular vesicles

Authors

Jesper Just[1]*

Yan Yan[2]*

Jean Farup[4,5]

Peter Sieljacks[3]

Mette Sloth[1]

Morten Venø[2]

Tingting Gu[1]

Frank Vincenzo de Paoli[6]

Jens Randel Nyengaard[7]

Rikke Bæk[8]

Malene Møller Jørgensen[8]

Jørgen Kjems[2, 2a]

Kristian Vissing[3]

Kim Ryun Drasbek[1*]

Supplementary Table S1: EVarray antibodies

Antibody target	Clone (if available)	Supplier
CD11a	HI111	Ab biotec, CA, USA
EGFR		Antibodies-online.com, Germany
CD146	P1H12	Abcam, UK
Hsp90	IGF1	Abcam, UK
p53	pAb240	Abcam, UK
Flotillin-1		Abcam, UK
LRP-1		Abcam, UK
N-Cadherin	8C119	Abcam, UK
CD63		AbD serotec /Bio-Rad, CA, USA
CD9		Ancell, MN, USA
CD81		Ancell, MN, USA
CD3	Hit3a	BD Biosciences, CA, USA
CD28	L293	BD Biosciences, CA, USA
CD49d	L25	BD Biosciences, CA, USA
CD56	3G8	BD Biosciences, CA, USA
CD25	M-A251	BD Biosciences, CA, USA
TGFβ1		BD Biosciences, CA, USA
Alix	3A9	Biolegend, CA, USA
VEGFR2	7D4-6	Biolegend, CA, USA
HER2	29D8	Cell Signalling, MA, USA
ICAM-1	R6.5	eBioscience, CA, USA
Annexin V		R&D Systems, MN, USA
CD19	4G7-2E3	R&D Systems, MN, USA
CD4	34930	R&D Systems, MN, USA
LAMP1		R&D Systems, MN, USA
LAMP2	H4A3	R&D Systems, MN, USA
CD82	423524	R&D Systems, MN, USA
CD106	HAE-2Z	R&D Systems, MN, USA
Thrombospondin		R&D Systems, MN, USA
CD31		R&D Systems, MN, USA
CD42b		R&D Systems, MN, USA
CD235a		R&D Systems, MN, USA
PD-L1	130021	R&D Systems, MN, USA
Hsp70	242707	R&D Systems, MN, USA
CD13	498001	R&D Systems, MN, USA
CD142	323514	R&D Systems, MN, USA
TNF RI		R&D Systems, MN, USA
TNF RII		R&D Systems, MN, USA
EpCam	0.N.277	Santa Cruz Biotechnologies, TX, USA
c-MET	016	Sino Biological Inc., China

List of antibodies included in the EVarray

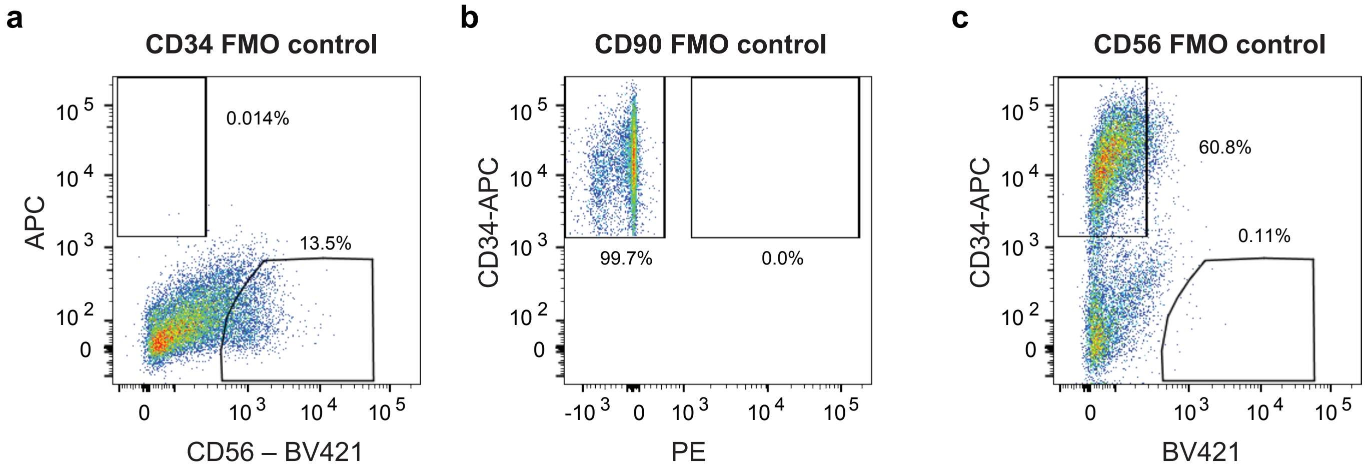

Supplementary Figure S1. Fluorescence minus one (FMO) controls.
To ensure specific gating of FAPs and MuSCs from a single cell suspension of skeletal muscle tissue, all gates were verified using a series of staining patterns that lacks just one of the fluorescent antibodies used for selection: CD34-APC (a), CD90-PE (b), and CD56-BV421 (c).

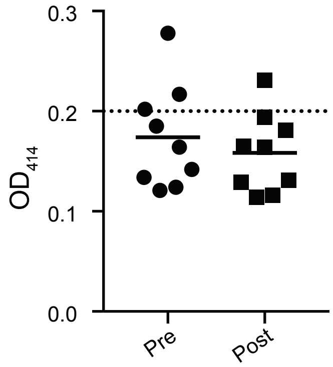

Supplementary Figure S2. Haemolysis levels of plasma samples.
The level of haemolysis in the BFRE plasma samples was assessed by measuring the absorbance of free haemoglobin at 414 nm. If OD414 exceeded 0.2, the plasma sample was considered to be affected by haemolysis.

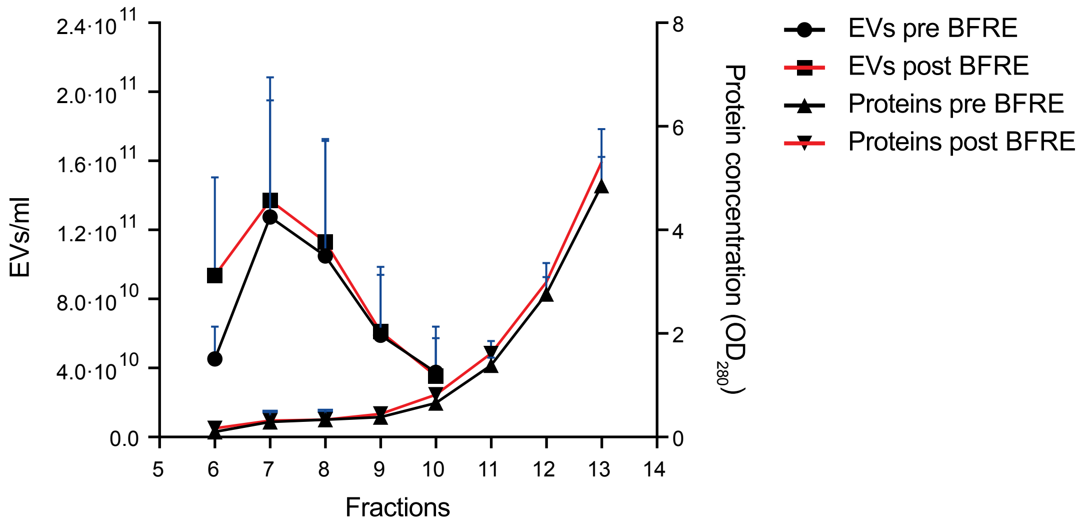

Supplementary Figure S3. Size exclusion chromatography elution profiles.
EV elution profile for a qEV size exclusion column when 1 ml plasma was loaded and fractions of 0.5 ml were collected. All pre and post BFRE plasma samples were analysed. A qNano Gold equipped with a NP100 Nanopore was used to determine size and concentration of EVs in the fractions eluted by SEC. EV samples were analysed under identical settings - the same diluent, stretch (45mm), pressure (10Pa) and voltage (0.7mV) as used for CPC100 calibration particles. To analyse protein contaminants, OD280 was measured on a nanodrop 2000. EVs devoid of protein contamination were present in fraction 6-9.

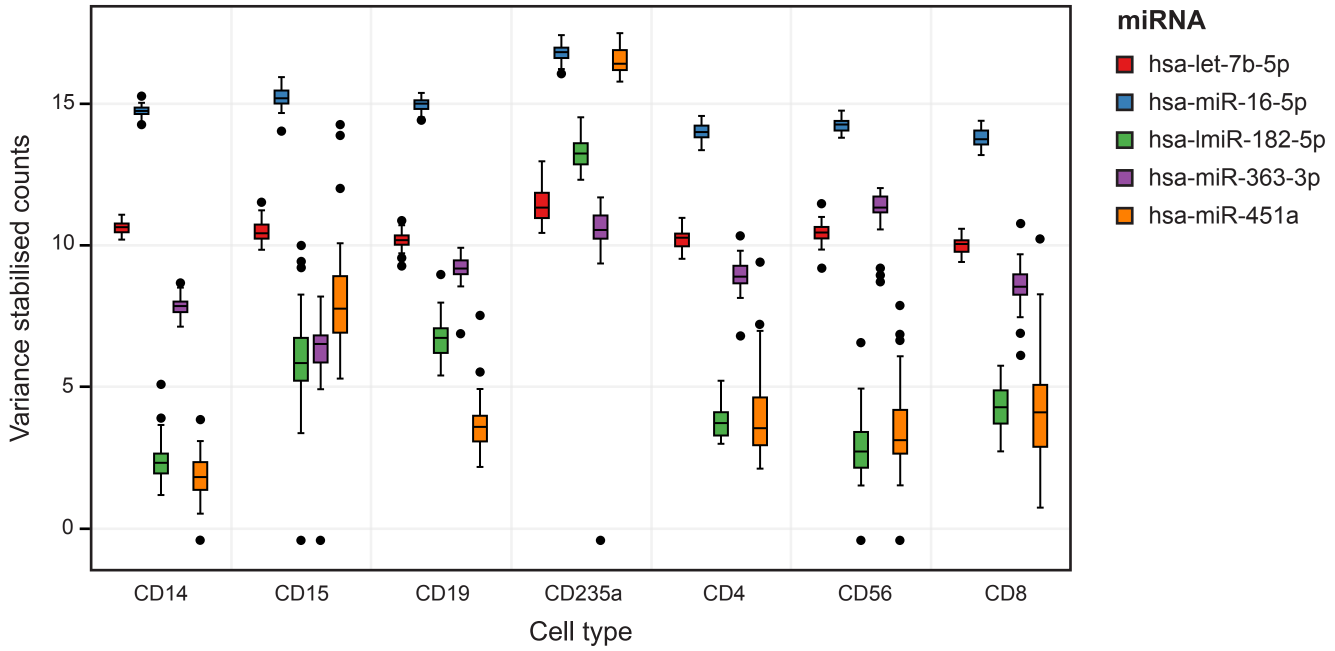

Supplementary Figure S4. Differentially expressed miRNAs prevalence in blood cell types.
The expression of the five up-regulated differentially expressed BFRE EV miRNAs in seven types of human peripheral blood cells. The plot was created using the interactive web tool: http://134.245.63.235/ikmb-tools/bloodmiRs. Most of the up-regulated miRNAs, especially miR-182-5p and miR-451a, were enriched in CD235a positive cells, while miR-16-5p was expressed in all cell types.

Supplementary Table S2. Gene targets of the differentially expressed EV miRNAs.

Gene target	Gene targeting miRNAs	Gene target	Gene targeting miRNAs	Gene target	Gene targeting miRNAs
NUFIP2	8	HSPA1B	4	WEE1	4
CCND2	6	HSPA8	4	SOCS3	4
E2F3	6	LBR	4	SOCS5	4
IGF1R	6	PRIM1	4	MORF4L1	4
MYC	6	MAPK1	4	ABHD2	4
hsa-mir-451a	5	SP1	4	SF3B3	4
CDK6	5	THBS1	4	RACGAP1	4
CDKN1A	5	TIAM1	4	TMEM138	4
SKI	5	TRAPPC10	4	MRPS10	4
SON	5	ZNF207	4	GPAM	4
BTG2	5	SOCS1	4	ZMAT3	4
NCOA3	5	WASL	4	WNK3	4
TNFRSF10B	5	MAP7	4	ATP13A3	4
ZNF460	5	ZNF264	4	ORAI2	4
TNRC6B	5	QKI	4	DCTN5	4
DICER1	5	ZBTB5	4	ACBD5	4
CBX5	5	LPGAT1	4	ARHGAP12	4
KPNA6	5	CDIPT	4	RAB3IP	4
SZRD1	5	TGOLN2	4	PTPDC1	4
YOD1	5	RNF44	4	CXorf38	4
LZIC	5	CPEB3	4	DYNC1LI2	4
SLC30A7	5	TMEM2	4	MBNL1	4
BCL2	5	TES	4	MDM2	4
TP53	5	AGO2	4	MMP2	4
RECK	5	SLC38A2	4	PKNOX1	4
TMEM245	5	TXLNG	4	RAB5B	4
PDCD4	5	RAP2C	4	ATXN1	4
KLHL15	5	NUCKS1	4	SOX4	4
CREB1	5	ZNF644	4	ZNF35	4
IPP	5	PPP1R15B	4	DEGS1	4
PTEN	5	YTHDC1	4	CLOCK	4
BCL2L11	5	GRPEL2	4	FRS2	4
SLC7A11	5	ZNF417	4	SGTB	4
MYLIP	5	AGO3	4	USP28	4
ACTB	4	FOXK1	4	GID4	4
AMD1	4	PGM2L1	4	PANK3	4
CCND1	4	CFL2	4	GATAD2B	4
BCL7A	4	DDX3X	4	FOXO3	4
EEF1A1	4	ITPR1	4	GK5	4
EIF4A2	4	TIMP3	4		
HMGB1	4	VEGFA	4		

The differentially expressed EV miRNAs were used as input for the miRnet database that integrate high-quality miRNA-target interaction from several databases. Genes targeted by more than three miRNAs were included in the table.
